# Physioxia: a more effective approach for culturing human adipose-derived stem cells for cell transplantation

**DOI:** 10.1186/s13287-018-0891-4

**Published:** 2018-05-24

**Authors:** Chang Chen, Qi Tang, Yan Zhang, Mei Yu, Wei Jing, Weidong Tian

**Affiliations:** 1 State Key Laboratory of Oral Diseases, National Clinical Research Center for Oral Diseases, West China Hospital of Stomatology, Sichuan University, Chengdu, People’s Republic of China; 2 National Engineering Laboratory for Oral Regenerative Medicine, West China Hospital of Stomatology, Sichuan University, Chengdu, People’s Republic of China; 3 Department of Oral and Maxillofacial Surgery, West China Hospital of Stomatology, Sichuan University, Chengdu, People’s Republic of China

**Keywords:** Physioxia, Adipose-derived stem cells, Cell survival, Culture approach, Cell therapy

## Abstract

**Background:**

Although typically cultured at an atmospheric oxygen concentration (20–21%), adipose-derived stem cells (ASCs) reside under considerable low oxygen tension (physioxia) in vivo. In the present study, we explored whether and how physioxia could be a more effective strategy for culturing ASCs for transplantation.

**Methods:**

After isolation, human ASCs were cultured under physioxia (2% O_2_) and hyperoxia (20% O_2_) until assayed. WST-8, Transwell, tube formation, β-galactosidase staining, and annexin V-FITC/PI assays were used to evaluate cell proliferation, migration, angiogenesis, senescence, and apoptosis, respectively. Survivability was determined by an ischemia model in vitro and nude mouse model in vivo, and the underlying metabolic alterations were investigated by fluorescence staining, flow cytometry, and real-time polymerase chain reaction.

**Results:**

Compared with those in the hyperoxia group, cells in the physioxia group exhibited increased proliferation, migration, and angiogenesis, and decreased senescence and apoptosis. The increased survival rate of ASCs cultured in physioxia was found both in ischemia model in vitro and in vivo. The underlying metabolic reprogramming was also monitored and showed decreased mitochondrial mass, alkalized intracellular pH, and increased glucose uptake and glycogen synthesis.

**Conclusions:**

These results suggest that physioxia is a more effective environment in which to culture ASCs for transplantation owing to the maintenance of native bioactivities without injury by hyperoxia.

## Background

Since first isolated in 1964 [1], human adipose-derived stem cells (ASCs) have garnered increasing attention [2]. Especially in the recent two decades, after the discovery of their stemness in 2001 [3], a growing body of research has indicated that ASCs possess properties of repair and regeneration, which include angiogenesis [4], multilineage differentiation [5], immunosuppression [6], and homing to ischemic tissues [7]. Consequently, there is great interest in and demand for utilizing ASCs in several clinical applications, such as osteoarthritis, heart failure treatment and wound healing, according to the clinicaltrials.gov database.

However, there are still several problems to resolve, such as the donor choice [8], therapeutic safety [9], and standard protocol for expanding ASCs [10]; among these problems, the most suitable strategy for culturing and expanding ASCs in vitro has been continuously studied. Several factors should be considered, such as the culture medium, serum replacements, and seeding density [11]. However, there is an extremely appropriate standard to which can be referred, the stem cell niche, which is the surrounding microenvironment and intrinsic factors that control the self-renewal and differentiation of stem cells [12, 13].

A distinct difference between “standard culture conditions” and the ASC niche is the oxygen level [14]. Cell culture is typically performed at an atmospheric O_2_ concentration (20–21%), i.e., the normoxia recognized by most researchers. However, there is a “mental shortcut” [15] neglecting the fact that the normoxia of 20–21% O_2_ reflects the pathology of humans or animals, while the practical oxygen concentration of the ASC niche is lower, at 2% [16], which is called physioxia [17]. In other words, atmospheric normoxia represents a hyperoxic state for ASCs.

Many biological alterations occur when culturing such cells under hyperoxia (atmospheric normoxia), particularly with respect to metabolism [18], generating changes in cell proliferation [19] and differentiation [20], among others [21]. Underlying these discrepancies is the impact of hypoxia-inducible factor 1 (HIF-1), which is degraded at O_2_ levels over 5% [15].

By comparison, previous studies have commonly used physioxia at 2% O_2_ to culture ASCs [22–24] as a transitory approach to increase the expansion and angiogenesis of ASCs rather than as a culture standard through the entire in vitro period, except for some studies [25–29]; yet, these studies did not examine the angiogenesis or survival of ASCs under an ischemic environment. Thus, the aim of the present study was to explore the superiority of physioxia (2% O_2_) compared with hyperoxia (20% O_2_) throughout the in vitro culture of ASCs by examining discrepancies in proliferation, migration, senescence, apoptosis, angiogenesis, and survivability, as well as the underlying mechanism.

## Methods

### Cell isolation and culture

Subcutaneous adipose tissue was collected from the abdomen of four healthy females (age, 25 ± 5 years, body mass index [BMI]: 19–22) after obtaining their consent. After washing with phosphate-buffered saline (PBS), the tissue was minced and digested with 0.2% collagenase (Sigma-Aldrich, St. Louis, MO, USA)/PBS for 40 min at 37 °C. The mixture was washed with PBS and centrifuged at 1000 rpm for 5 min, and the remaining pellet was cultured in α-modified Eagle’s medium (α-MEM; HyClone, GE Healthcare, Marlborough, MA, USA), 10% fetal bovine serum (FBS; Gibco, San Jose, CA, USA), 100 IU penicillin, and 100 mg/mL streptomycin (Solarbio, Beijing, China). Cells in the physioxia group were cultured with 2% O_2_ (using a modular chamber, Sanyo, Osaka, Japan) and 5% CO_2_ at 37 °C (physioxia ASCs, P-ASCs) until further analysis in the following tests at passage 3, with 20% O_2_ and 5% CO_2_ at 37 °C as a control (hyperoxia ASCs, H-ASCs). Cells from different donors were mixed at passage 2 to explore the general effect on ASCs.

### Cell characterization

#### Flow cytometric analysis

Flow cytometry was used to analyze the surface markers of the ASCs. After detaching, 1 × 10^5^ cells were incubated with PE- or FITC-conjugated antibodies against CD31, CD34, CD73, CD90, CD105, and HLA-DR for 30 min at 4 °C. All antibodies were obtained from Abcam Biotechnology (Abcam, Cambridge, MA, USA). The cells were then analyzed using a BD Accuri™ C6 flow cytometer (BD Biosciences, San Jose, CA, USA).

#### Adipogenesis

The cells were seeded onto six-well plates. After reaching 80% confluence, the culture medium was changed to α-MEM supplemented with 10% FBS, 1 mmol/L dexamethasone (Sigma-Aldrich, St. Louis, MO, USA), 10 mmol/L insulin (Sigma-Aldrich, St. Louis, MO, USA), 200 mmol/L indomethacin (Sigma-Aldrich, St. Louis, MO, USA) and 0.5 mmol/L 3-isobutyl-1-methylxanthine (IBMX; Sigma-Aldrich, St. Louis, MO, USA) for 7 days. Lipid clusters were stained with oil red O.

### Western blotting

Western blotting was performed as previously described [30], with slight modifications. After being dissolved in radioimmunoprecipitation assay (RIPA) buffer (KeyGEN, Nanjing, Jiangsu, China), 30 μg of protein, as detected by bicinchoninic acid (BCA) assay, was separated on a 10% polyacrylamide gel and blotted onto a polyvinylidene fluoride (PVDF) membrane. The membrane was blocked with 5% skim milk and then treated with primary antibodies against HIF-1 (1:1000, 14,179, Cell Signaling Technology, Beverly, MA, USA) and β-actin (1:1000, ab3280, Abcam, Cambridge, MA, USA) overnight at 4 °C, followed by 1 h of incubation with horseradish peroxidase (HRP)-conjugated secondary antibodies at room temperature. The signals were detected with Amersham ECL Select Western Blotting Detection Reagent (GE, Waukesha, WI, USA) according to the manufacturer’s protocol. The signals were visualized using an ImageQuant LAS 4000 mini (GE, Waukesha, WI, USA).

### WST-8

Cell Counting Kit 8 (WST-8; Dojindo, Kumamoto, Japan) was used to determine the proliferation of P-ASCs and H-ASCs. The cells (1 × 10^3^) were seeded onto 96-well plates, and after 1, 2, 3, 4, 5, 6, and 7 days, the culture medium was replaced with 100 μL of WST-8 dye solution (90 μL of α-MEM with 10 μL WST-8) for 2 h at 37 °C. Subsequently, the medium was discarded, and the absorbance at 450 nm was detected using a spectrophotometer (Multiskan GO, Thermo Fisher Scientific, Waltham, MA, USA).

### Cell doubling curve

ASCs were seeded onto six-well plates at a concentration of 3× 10^4^ per well. The cells were collected at the indicated time points (1, 2, 3, 4, 5, 6, and 7 days), and the cell numbers were measured using an Automated Cell Counter (Bio-Rad, Hercules, CA, USA).

### Determination of reactive oxygen species (ROS), mitochondrial mass, and glucose uptake

The ROS level, mitochondrial mass and glucose uptake were determined by staining with 1 μM dihydrodichlorofluorescein diacetate (H_2_DCFDA, Sigma-Aldrich, St. Louis, MO, USA), 10 nM nonyl acridine orange (NAO, Sigma-Aldrich, St. Louis, MO, USA) and 150 μM 2-[N-(7-nitrobenz-2-oxa-1,3-diazol-4-yl) amino]-2-deoxy-D-glucose (2-NBDG, Life Technologies, Gaithersburg, MD, USA), respectively, for 30 min at 37 °C. The results were acquired by fluorescence microscopy (Olympus, Hamburg, Germany) and BD Accuri™ C6 (BD Biosciences, San Jose, CA, USA) with a minimum of 5000 events per sample. ROS inhibition was produced using 100 μM butylated hydroxyanisole (BHA, Sigma-Aldrich, St. Louis, MO, USA).

### Transwell assay

After incubation in serum-free medium for 24 h, 1 × 10^5^ cells were transferred to the upper chamber of a Transwell (Corning, Corning, NY, USA). Medium containing 10% FBS was added to the lower chamber as a chemoattractant. After 24 h, nonmigratory cells in the upper chamber were removed. The migrated cells were fixed with 4% paraformaldehyde for 30 min, followed by 0.1% crystal violet (Sigma-Aldrich, St. Louis, MO, USA) staining for 15 min. After images were captured, the crystal violet in the cells was extracted by 10% acetic acid for 15 min, and the absorbance at 600 nm was measured using a spectrophotometer (Multiskan GO, Thermo Fisher Scientific, Waltham, MA, USA).

### Cell senescence

We assayed the ASCs for senescence-associated β-galactosidase (SA-β-Gal) activity using a Senescence β-Galactosidase Staining Kit (Beyotime, Shanghai, China) according to the manufacturer’s instructions. The SA-β-Gal^+^ area was calculated by ImageJ (NIH, Bethesda, MD, USA) using the ratio of Periodic acid-Schiff (PAS)^+^ area to the total area of the image.

### Cell apoptosis

ASC apoptosis was measured using an Annexin V-FITC/PI Apoptosis Detection Kit (KeyGEN, Nanjing, Jiangsu, China) according to the manufacturer’s instructions. Flow cytometry was conducted using the BD Accuri™ C6 flow cytometer (BD Biosciences, San Jose, CA, USA).

### Tube formation assay

ASCs (2 × 10^5^) were seeded onto 96-well plates coated with Matrigel (Corning, Corning, NY, USA). After incubation at 37 °C for 6 h, the cells were imaged under a microscope (Olympus, Hamburg, Germany). The images were quantified using ImageJ (NIH, Bethesda, MD, USA).

### Real-time polymerase chain reaction (RT-PCR)

RNA was extracted using RNAiso Plus (TaKaRa Biotechnology, Dalian, Liaoning, China) according to the manufacturer’s instructions, followed by cDNA synthesis using a First Strand cDNA Synthesis Kit (Thermo Fisher Scientific, Waltham, MA, USA). Quantitative RT-PCR was performed using the Eco Real-Time PCR System (Illumina, San Diego, CA, USA) and SYBR Premix Ex Taq (TaKaRa Biotechnology, Dalian, Liaoning, China) with the following conditions: 2 min at 95 °C, followed by 40 cycles of 5 s at 95 °C and 30 s at 60 °C. The relative expression levels were calculated by the 2^-ΔΔct^ method and normalized to the housekeeping gene HPRT. The primer sequences are displayed in Table 1.

**Table 1:** Primer sequences

Gene	Forward (5′ to 3′)	Reverse (5′ to 3′)
HPRT	CCTGACCAAGGAAAGCAAAG	GACCAGTCAACAGGGGACAT
VEGF	AGGGAAGAGGAGGAGATGAG	GCTGGGTTTGTCGGTGTT
VEGFR2	CTGGCTACTTCTTGTCATCATCCTACG	TGGCATCATAAGGCAGTCGTTCAC
vWF	ACCTTGGTCACATCTTCACATTCACTC	AAGTCATTGGCTCCGTTCTCATCAC
BNIP3	AGGGCTCCTGGGTAGAACTG	ACTCCGTCCAGACTCATGCT
COX4I1	GCCATGTTCTTCATCGGTTT	CATCCTCTTGGTCTGCTTGG
COX4I2	CCCTACACCAACTGCTATGC	CTTCCCTTCTCCTTCTCCTTC
PDK1	AATCACACAGACGCCTAGCA	CATCCTCTTGGTCTGCTTGG
LDHA	ATCTTGACCTACGTGGCTTGGA	CCATACAGGCACACTGGAATCTC
MCT4	ATCTGCTTTGCCATCTTTGC	GTCCAGAAAGGACAGCCATC
NHE2	TTCATGCCACGGATAAATGA	TTCTCTTCAGGCCAGCAAAT
NHE3	AGGTCCATGTCAACGAGGTC	ACTATGCCCTTCACGCAGTC
CAR9	GTCTCGCTTGGAAGAAATCG	ACAGGGCGGTGTAGTCAGAG
GLUT1	CATAGCCACCTCCTGGGATA	AATCACACAGACGCCTAGCA
GLUT3	GCACATAGCTATCAAGTGTGC	AGTGAGAAATGGGACCCTGC
PGM	TGGAAATACGGAATGCTGAA	GCTGCCTTTGATGGAGATG
GYS1	ACCCACCTTGTTAGCCACCT	AACCGCACTTTGTCCATGTC
PYGL	CCAAAGCAGCCACATCATC	GCCCTAACTATCGGGACCAT

### Cell survival

The survival assay was conducted as previously described [31]. Briefly, four harsh conditions (ischemic [1% O_2_, pH 6.4 and 0.56 μM glucose], hypoxic [1% O_2_, pH 7.4 and 5.6 μM glucose], acidic [20% O_2_, pH 6.4 and 5.6 μM glucose], and nutrient-depleted [20% O_2_, pH 7.4 and 0.56 μM glucose] environments) were generated using a modular chamber (Sanyo, Osaka, Japan) and N-2-hydroxyethylpiperazine-N′-2-ethanesulphonic acid-buffered Tyrode’s solution. After incubating the ASCs for 24 h on 96-well plates (1 × 10^4^ per well), live/dead staining and WST-8 were applied to determine the survival of the P-ASCs and H-ASCs.

### Intracellular pH detection

ASCs (1 × 10^4^) on 96-well plates were stained with 5 μM 2′,7′-bis-(2-carboxyethyl)-5-(and-6)-carboxyfluorescein, acetoxymethyl ester (BCECF-AM, Millipore, Billerica, MA, USA) for 30 min at 37 °C. A Multimode Reader (Thermo Fisher Scientific, Waltham, MA, USA) was employed to measure the intracellular pH at excitation and emission wavelengths of 500 nm and 530 nm, respectively. A calibration curve was produced by dyeing ASCs with 5 μM BCECF-AM for 30 min, with subsequent application of an Intracellular pH Calibration Buffer Kit (Thermo Fisher Scientific, Waltham, MA, USA) under different pH values (4.5, 5.5, 6.5, and 7.5) in the presence of 10 μM K^+^/H^+^ ionophore nigericin (Thermo Fisher Scientific, Waltham, MA, USA).

### PAS staining

We used PAS staining to explore the different expression levels of glycogen in P-ASCs and H-ASCs. Cells on six-well plates were fixed with 4% paraformaldehyde and incubation for 5 min with 0.5% periodic acid (Solarbio, Beijing, China), followed by Schiff’s reagent for 15 min. After the cells were imaged, the PAS^+^ area was quantified by ImageJ (NIH, Bethesda, MD, USA).

### Extracellular lactate assay

The lactate content of the culture medium was measured using a Lactate Assay Kit (KeyGEN, Nanjing, Jiangsu, China) according to the manufacturer’s protocol.

#### In vivo experiment

Fibrin gel and TdT-mediated dUTP-biotin nick end labeling (TUNEL) assays were conducted as previously described [31]. The fibrin gel was composed of 25 mg/mL fibrinogen, 20 mM CaCl_2_, and 2.5 U/mL thrombin. The cells (1 × 10^6^) were mixed with 80 μL of fibrin gel and subcutaneously transplanted into the dorsum of nude mice under deep anesthesia. After 24, 48 and 72 h, the constructs were removed and immediately fixed with 4% paraformaldehyde for paraffin embedding. Subsequently, 5-μm-thick sections were cut and subjected to TUNEL assay using an In Situ Cell Death Detection Kit (KeyGEN, Nanjing, Jiangsu, China) to measure the ASC death. The number of TUNEL^+^ cells was analyzed using Image-Pro Plus. Animal studies were conducted according to the protocol approved by the Ethics Committee of the State Key Laboratory of Oral Diseases, West China School of Stomatology, Sichuan University, China.

### Statistics

Data were analyzed with GraphPad Prism 5.02 (GraphPad Software, San Diego, CA, USA) and are expressed as the mean ± standard deviation. Unpaired Student’s *t* tests were performed, and statistical significance was considered at *P* < 0.05. At least three replicates were analyzed in each experiment.

## Results

### Identification of P-ASCs and H-ASCs

**Fig. 1:**
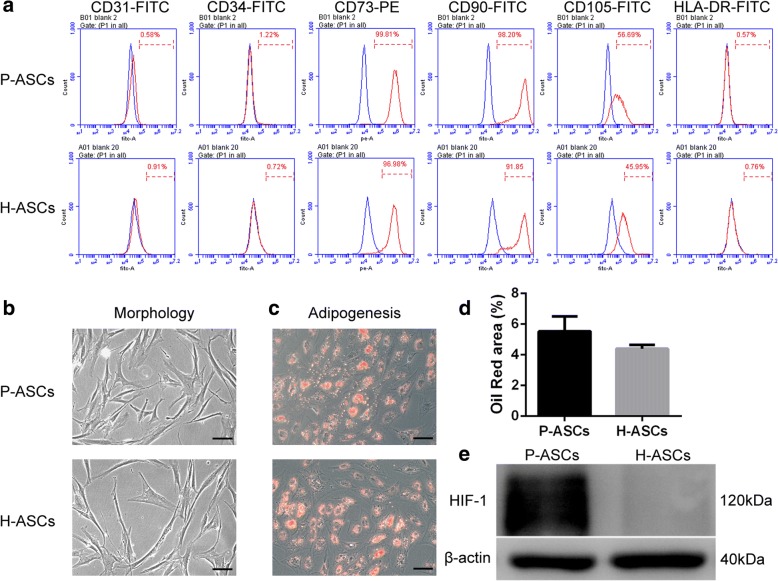
Characterization of P-ASCs and H-ASCs. Human ASCs were cultured under physioxia (2% O_2_, P-ASCs) or hyperoxia (20% O_2_, H-ASCs) for the entire in vitro period until assayed at passage 3. **a** Flow cytometry was applied to demonstrate the immunophenotype of P-ASCs and H-ASCs. **b** Morphology of ASCs cultured in α-modified Eagle’s medium (α-MEM) with 10% fetal bovine serum (FBS). **c** After inducing adipogenesis for 7 days, lipid clusters were stained by oil red O. **d** Oil red O staining was quantified by the ratio of oil red O^+^ area to total image area for three fields. Data are presented as the mean ± SD, *P > 0.05. **e** Western blot of hypoxia-inducible factor 1 (HIF-1) and β-actin (reference gene) expression. Scale bar = 50 μm. *ASCs* adipose-derived stem cells, *H-ASCs* hyperoxia ASCs, *P-ASCs* physioxia ASCs

Flow cytometric analysis indicated that the P-ASCs (physioxia ASCs) and H-ASCs (hyperoxia ASCs) were positive for CD73, CD90, and CD105 and negative for CD31, CD34, and HLA-DR (Fig. 1a). Both the P-ASCs and H-ASCs exhibited a typical spindle-shaped morphology (Fig. 1b) and adipogenic ability (Fig. 1c and d). Compared with the H-ASCs, the P-ASCs exhibited upregulated HIF-1 protein expression, as determined by Western blotting (Fig. 1e).

### Physioxia enhanced ASC proliferation and migration through ROS upregulation

**Fig. 2:**
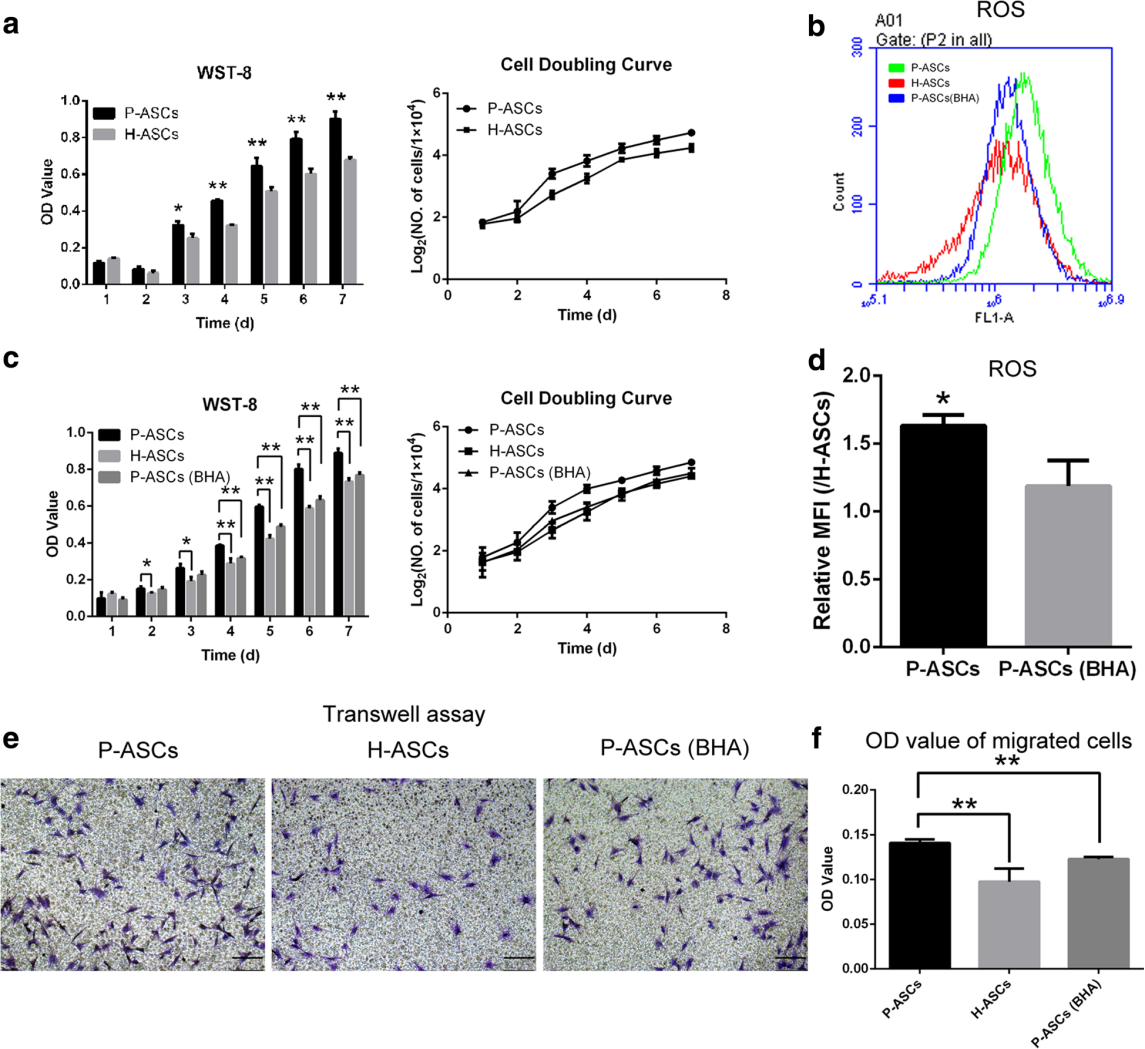
Physioxia enhanced ASC proliferation and migration through ROS upregulation. **a** The proliferation of P-ASCs and H-ASCs measured by WST-8 and cell doubling curves. **b** and **d** P-ASCs were treated with 100 μM BHA to inhibit ROS, as detected by flow cytometry. The relative MFI was quantified by the ratio of the MFI for P-ASCs and P-ASCs (BHA) to that of H-ASCs. **c** The proliferation of P-ASCs, H-ASCs and P-ASCs (BHA) measured by WST-8 and cell doubling curves. **e** Transwell assays were used for determining cell migration, and the migrated cells were stained by 0.1% crystal violet. **f** The crystal violet in migrated cells was extracted by 10% acetic acid, and the optical density values were determined. The cell doubling curve was produced by dividing the cell number by 10^4^ and then transforming the values to log_2_. Data are presented as the mean ± SD, *P < 0.05, **P < 0.01, Student’s *t* tests, scale bar = 100 μm. *ASCs* adipose-derived stem cells, *BHA* butylated hydroxyanisole, *H-ASCs* hyperoxia ASCs, *MFI* mean fluorescence intensity, *P-ASCs* physioxia ASCs, *ROS* reactive oxygen species

Using WST-8 and cell doubling curves, P-ASCs exhibited increased proliferation (Fig. 2a) accompanied by an increased ROS level (Fig. 2b and d). After ROS inhibition in P-ASCs by BHA (Fig. 2b, d), the enhanced P-ASC proliferation was decreased (Fig. 2c). Similarly, the Transwell assay (Fig. 2e, f) revealed reduced migration in H-ASCs and P-ASCs (BHA).

### Physioxia inhibited ASC senescence and apoptosis

**Fig. 3:**
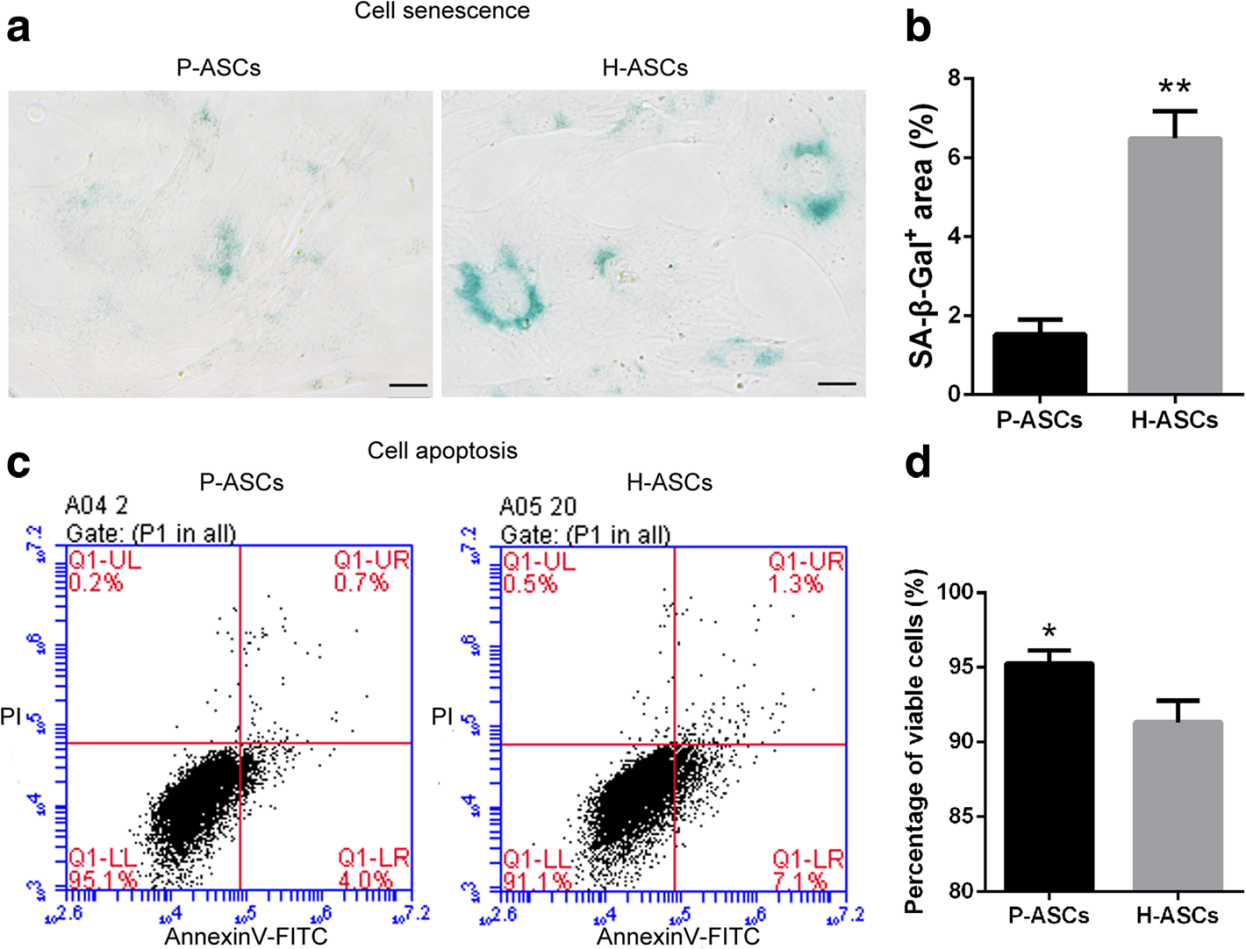
Physioxia inhibited ASC senescence and apoptosis. **a** Microscopy images of senescent cells shown by SA-β-Gal staining. **b** SA-β-Gal staining results were quantified by the ratio of SA-β-Gal^+^ area to the total image area for three fields. **c** Cell apoptosis measured by flow cytometry using annexin V-FITC/PI double staining. Q1-UL, mechanical error; Q1-UR, late apoptotic or necrotic cells; Q1-LL, viable cells; Q1-LR, early apoptotic cells. **d** The ratio of viable cells acquired from Q1-LL. Data are presented as the mean ± SD, * *P* < 0.05, ** *P* < 0.01, Student’s *t* tests, scale bar = 20 μm. *ASCs* adipose-derived stem cells, *H-ASCs* hyperoxia ASCs, *P-ASCs* physioxia ASCs, *SA-β-Gal* senescence-associated β-galactosidase

SA-β-Gal staining revealed that physioxia inhibited ASC senescence (Fig. 3a), with a significant difference in the SA-β-Gal^+^ area (1.53 ± 0.22% vs. 6.50 ± 0.40%, *P* < 0.01, Fig. 3b). Cell viability was significantly increased under physioxia compared with hyperoxia (95.27 ± 0.50% vs*.* 91.33 ± 0.85%, *P* < 0.05, Fig. 3c, d).

### Angiogenic activities of ASCs were promoted under physioxia

**Fig. 4:**
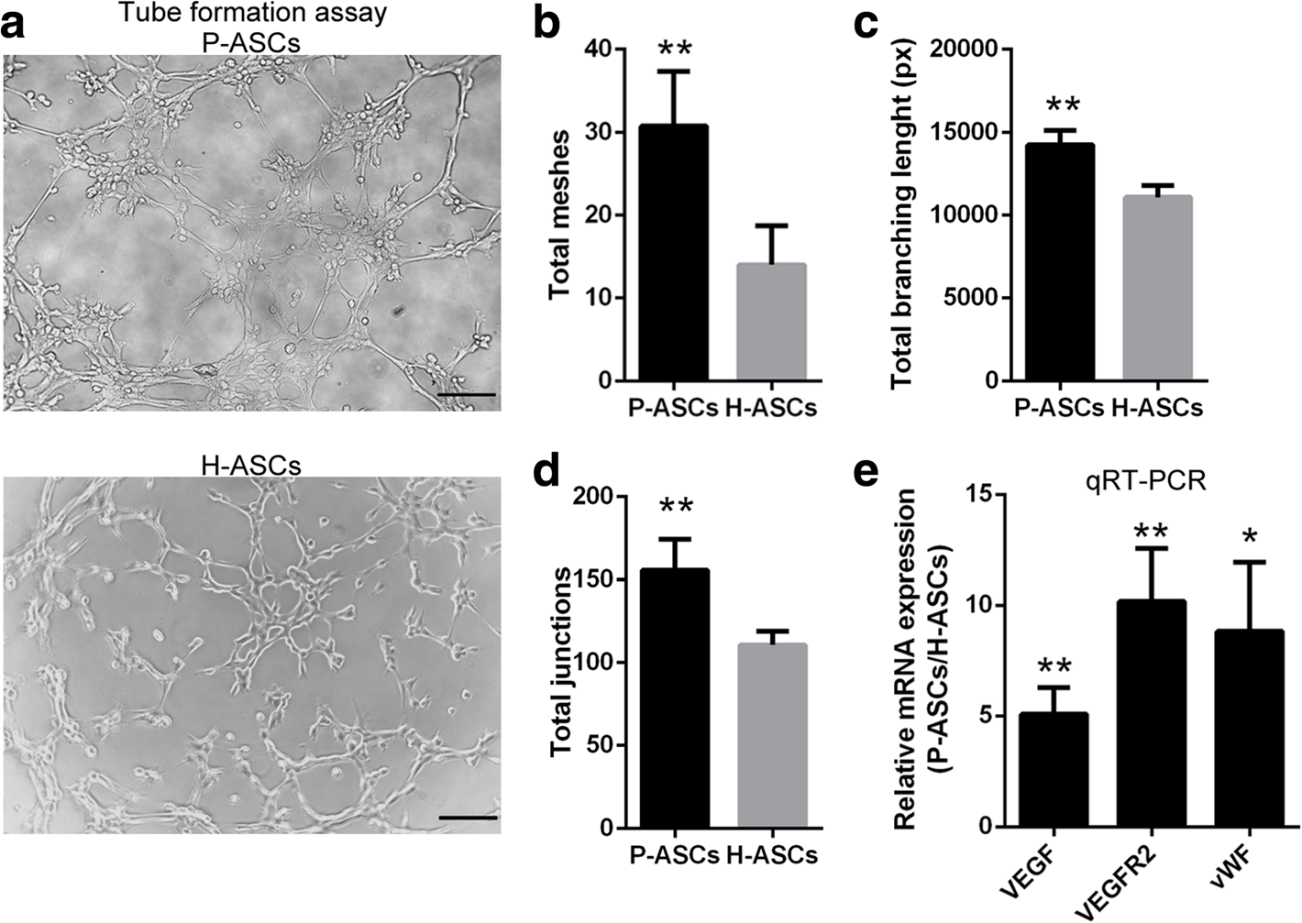
Physioxia promoted angiogenic ability of ASCs. ASCs (2 × 10^4^) were seeded onto 96-well plates coated with 50 μL of Matrigel and cultured for 6 h. **a** Mesh-like structures resulting from tube formation assay. **b**, **c** and **d** Total mesh, branching length, and junction values per field of view were quantified by ImageJ. Five fields were quantified. **e** Expression levels of mRNA encoding VEGF, VEGFR2, and vWF as measured by qRT-PCR. Data are presented as the mean ± SD, *P < 0.05 (P-ASCs/H-ASCs), **P < 0.01 (P-ASCs/H-ASCs), Student’s *t* tests, *n* = 3, scale bar = 100 μm. *ASCs* adipose-derived stem cells, *H-ASCs* hyperoxia ASCs, *P-ASCs* physioxia ASCs, *qRT-PCR* quantitative real-time polymerase chain reaction, *VEGF* vascular endothelial growth factor, *VEGFR2* vascular endothelial growth factor receptor 2, *vWF* von Willebrand factor

Tube formation induced by Matrigel was employed to examine the angiogenic activities of the cells. The P-ASCs generated more meshes than the H-ASCs (Fig. 4a), and statistical analysis revealed significantly increased total mesh (Fig. 4b), branching length (Fig. 4c) and junction (Fig. 4d) values for P-ASCs than for H-ASCs (2.20-, 1.29-, and 1.41-fold greater, respectively). RT-PCR showed increased expression of the angiogenic genes vascular endothelial growth factor (VEGF), vascular endothelial growth factor receptor 2 (VEGF-R2) and von Willebrand factor (vWF) (Fig. 4e) in P-ASCs.

### Survival of P-ASCs was strengthened under ischemic condition

**Fig. 5:**
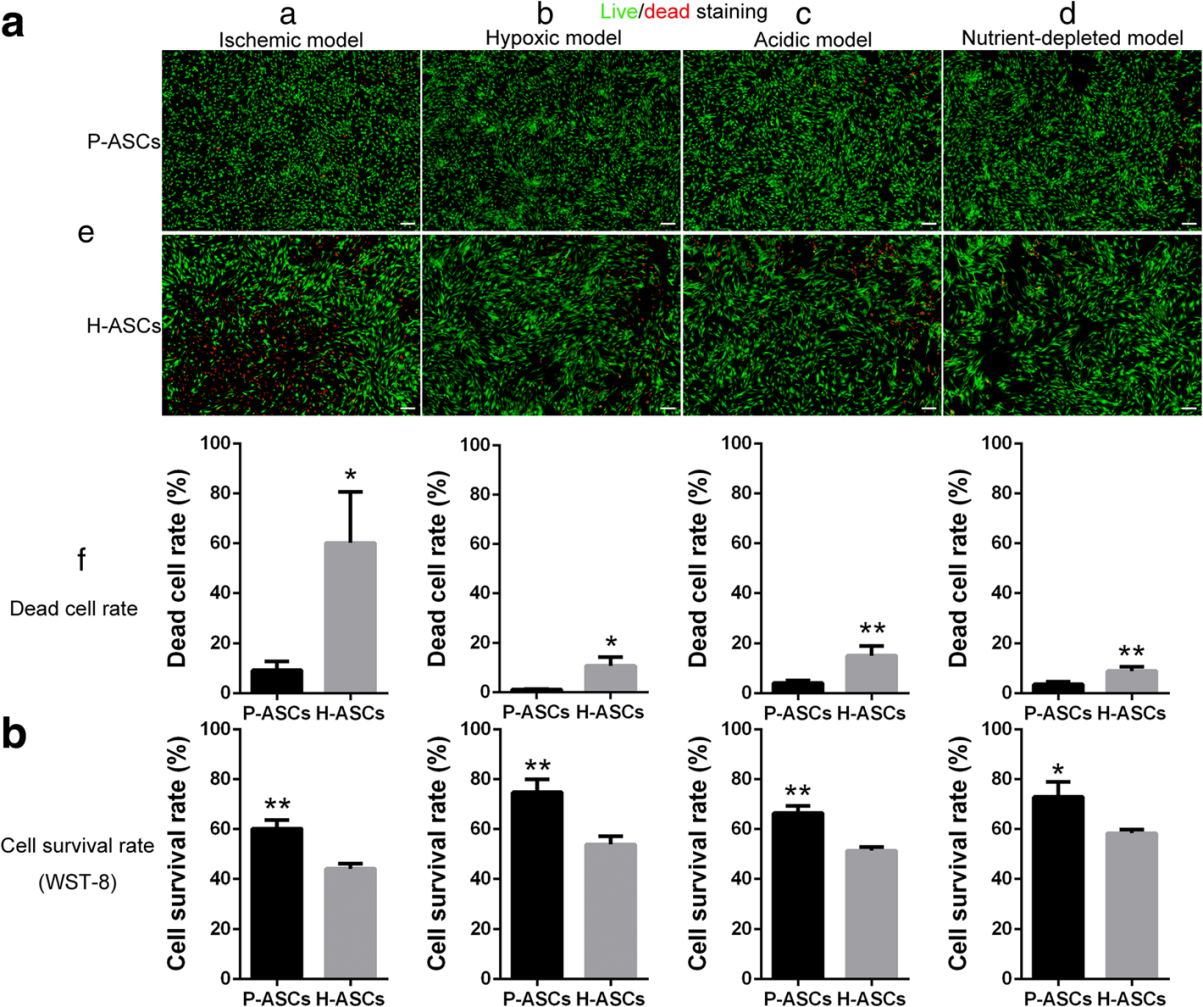
Physioxia improved ASC survivability under ischemic conditions. ASCs (1 × 10^4^) were seeded onto 96-well plates and incubated in four hostile environments for 24 h: (**a**) ischemic model, 1% O_2_, pH 6.4 and 0.56 μM glucose; (**b**) hypoxic model, 1% O_2_, pH 7.4 and 5.6 μM glucose; (**c**) acidic model, 20% O_2_, pH 6.4 and 5.6 μM glucose; (**d**) nutrient-depleted model, 20% O_2_, pH 7.4 and 0.56 μM glucose. (**A**) Fluorescent images showing the cell death rate by live/dead cell staining. The cell death rate was obtained by the ratio of dead cells to total cells. Three fields were quantified. (**B**) The cell survival rate was detected by WST-8 presented as the ratio of OD_24_ to OD_0_. Data are presented as the mean ± SD, *P < 0.05, **P < 0.01, Student’s *t* tests, scale bar = 200 μm. *ASCs* adipose-derived stem cells, *H-ASCs* hyperoxia ASCs, OD_0_, optical density value at 0 h, OD_24_, optical density value at 24 h, P-ASCs, physioxia ASCs

After incubation in an ischemic environment (Fig. 5a) for 24 h, P-ASCs showed increased survival (Fig. 5B) and decreased death rates (Fig. 5A). A minor but significant difference was also detected under the hypoxic (Fig. 5b), acidic (Fig. 5c), and nutrient-depleted conditions (Fig. 5d).

### Variations in mitochondrial and pH metabolism of ASCs under physioxia

**Fig. 6:**
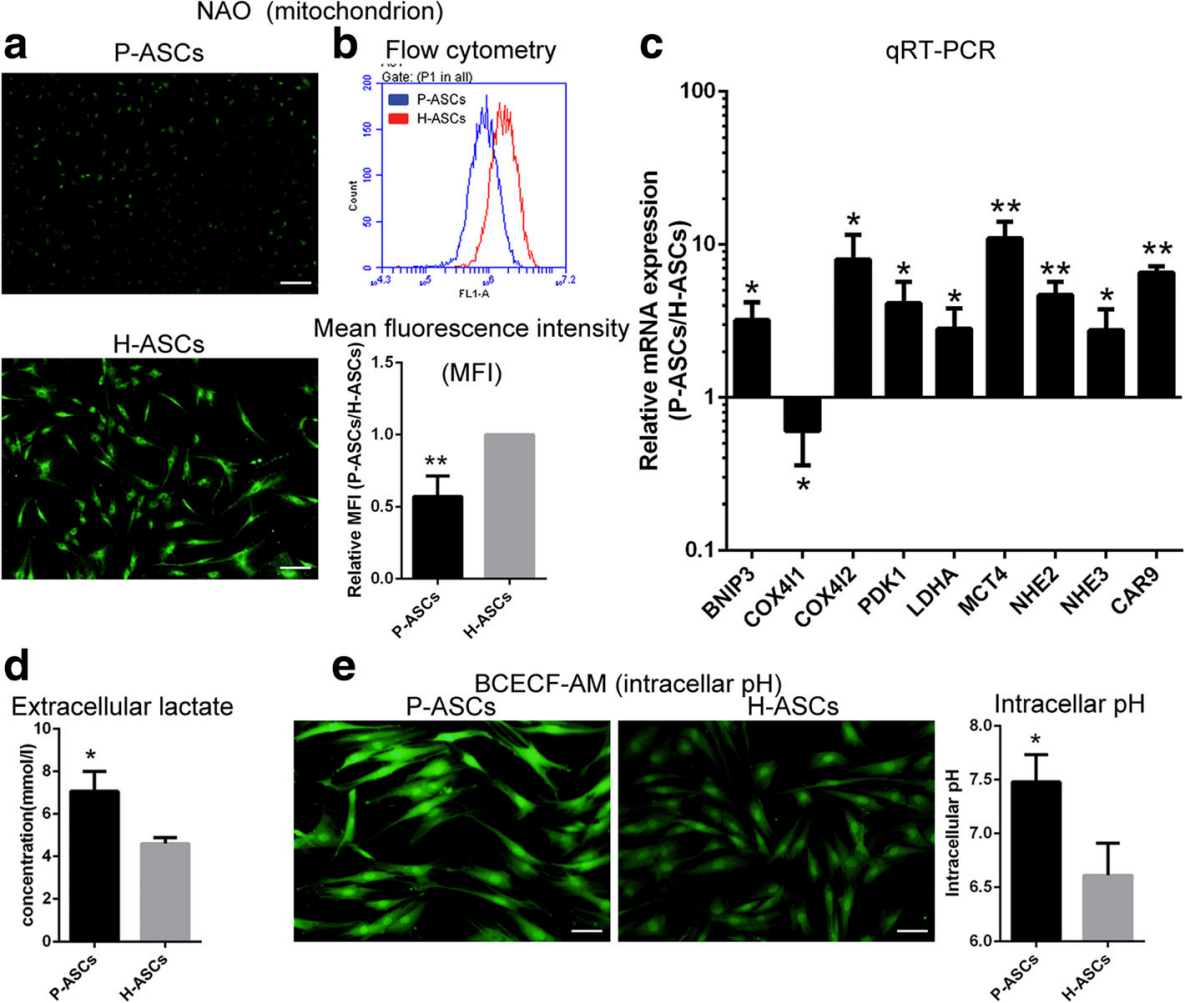
Variations in mitochondrial and pH metabolism of P-ASCs. **a** and **b** Fluorescent images and flow cytometry results of mitochondrial mass determined by staining ASCs with NAO; the relative MFI was determined as the MFI of P-ASCs versus that of H-ASCs. **c** Expression of HIF-1 target genes evaluated by qRT-PCR. **d** Extracellular lactate concentration of P-ASCs and H-ASCs. **e** Cells cultured in the acidic model for 24 h were stained with BCECF-AM to determine the intracellular pH. Data are presented as the mean ± SD, *P < 0.05 (P-ASCs/H-ASCs), **P < 0.01 (P-ASCs/H-ASCs), Student’s *t* tests, *n* = 3, scale bar = 100 μm. *ASCs* adipose-derived stem cells, *BCECF-AM* 2′,7′-bis-(2-carboxyethyl)-5-(and-6)-carboxyfluorescein, acetoxymethyl ester, *BNIP3* BCL2/adenovirus E1B 19 kDa protein-interacting protein 3, mitophagy regulator, *COX4I1* cytochrome c oxidase subunit 4 isoform 2, metabolic enzyme, *COX4I2* cytochrome c oxidase subunit 4 isoform 2, metabolic enzyme, *H-ASCs* hyperoxia ASCs, *LDHA* lactate dehydrogenase A, glycolysis, *MCT4* monocarboxylate transporter 4, lactate discharge, *NAO* nonyl acridine orange, *NHE2* sodium-hydrogen exchanger 2, H^+^ discharge, *NHE3* sodium-hydrogen exchanger 3, H^+^ discharge, *P-ASCs* physioxia ASCs, *PDK1* pyruvate dehydrogenase kinase 1, inactivating pyruvate dehydrogenase

By NAO staining, we measured a 43% decrease in the mitochondrial mass of P-ASCs (Fig. 6a, b), and the extracellular lactate concentration was much higher compared with that of H-ASCs (7.07 ± 0.54 vs*.* 4.60 ± 0.16, *P* < 0.05, Fig. 6d). Underlying these changes was the apparently upregulated mRNA expression of BCL2/adenovirus E1B 19 kDa protein-interacting protein 3 (BNIP3), cytochrome c oxidase subunit 4 isoform 2 (COX4I2), pyruvate dehydrogenase kinase 1 (PDK1) and lactate dehydrogenase A (LDHA), as detected by RT-PCR (Fig. 6c).

Cells were treated under acidic conditions (pH 6.4) for 24 h, and distinct alkalization in H-ASCs was determined by intracellular pH detection (7.48 ± 0.15 vs. 6.61 ± 0.17, *P* < 0.05, Fig. 6e). Additionally, the transcript levels of sodium-hydrogen exchangers (NHE2 and NHE3), carbonic anhydrase 9 (CAR9) and monocarboxylate transporter 4 (MCT4) were increased.

### Ascending glucose uptake and reserve in P-ASCs

**Fig. 7:**
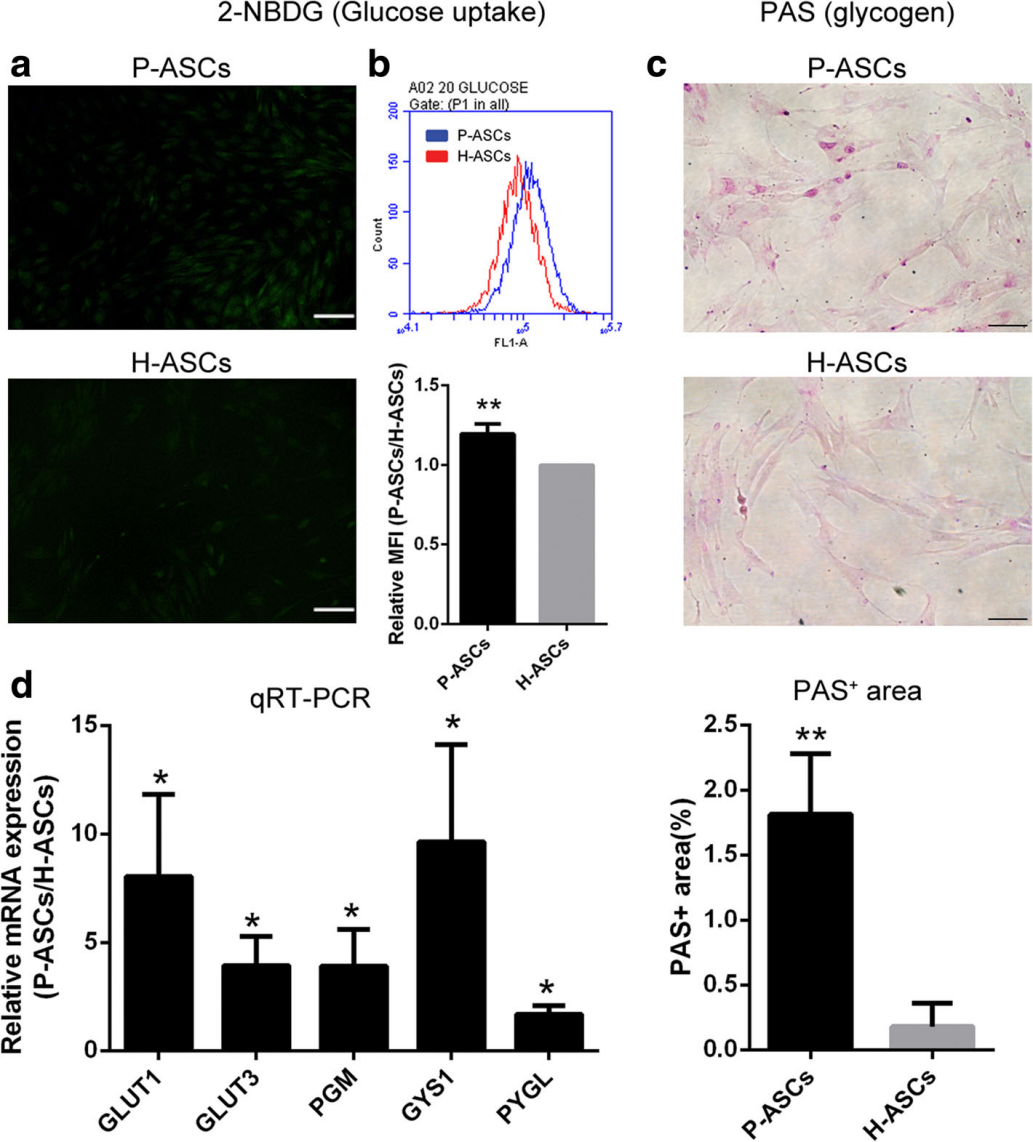
Increased glucose uptake and reserve of P-ASCs. **a** and **b** Fluorescent images and flow cytometry results of glucose uptake in ASCs determined by staining with 2-NBDG; the relative MFI was determined by MFI of P-ASCs versus that of H-ASCs. Scale bar = 100 μm. **c** Intracellular glycogen detected by PAS staining; the PAS^+^ area was calculated as the PAS^+^ area versus the total image area. Three fields were quantified. Scale bar = 50 μm. **d** Expression of HIF-1 target genes evaluated by qRT-PCR. Data are presented as the mean ± SD, *P < 0.05 (P-ASCs/H-ASCs), **P < 0.01 (P-ASCs/H-ASCs), Student’s *t* tests, *n* = 3. *2-NBDG* 2-[N-(7-nitrobenz-2-oxa-1,3-diazol-4-yl) amino]-2-deoxy-D-glucose, *ASCs* adipose-derived stem cells, *GLUT1* glucose transporter 1, glucose uptake, *GLUT3* glucose transporter 3, glucose uptake, *GYS1* glycogen synthase 1, glycogen synthesis, *H-ASCs* hyperoxia ASCs. *PAS* periodic acid-Schiff, *P-ASCs* physioxia ASCs, *PGM* phosphoglucomutase, glycogen synthesis, *PYGL* liver isoform of glycogen phosphorylase, glycogen breakdown

P-ASCs showed significantly increased glucose uptake, as measured by 2NBDG staining (1.20-fold greater, Fig. 7a, b), along with augmented mRNA levels of glucose transporters (GLUT1 and GLUT3), as demonstrated by RT-PCR (Fig. 7d). Increased glycogen reserves were found in P-ASCs, as detected by PAS staining (Fig. 7c), and the expression of glycogen synthesis (phosphoglucomutase [PGM] and glycogen synthase 1 [GYS1]) and breakdown genes (liver isoform of glycogen phosphorylase [PYGL]) were also upregulated (Fig. 7d).

### Increased survivability of P-ASCs *in vivo*

**Fig. 8:**
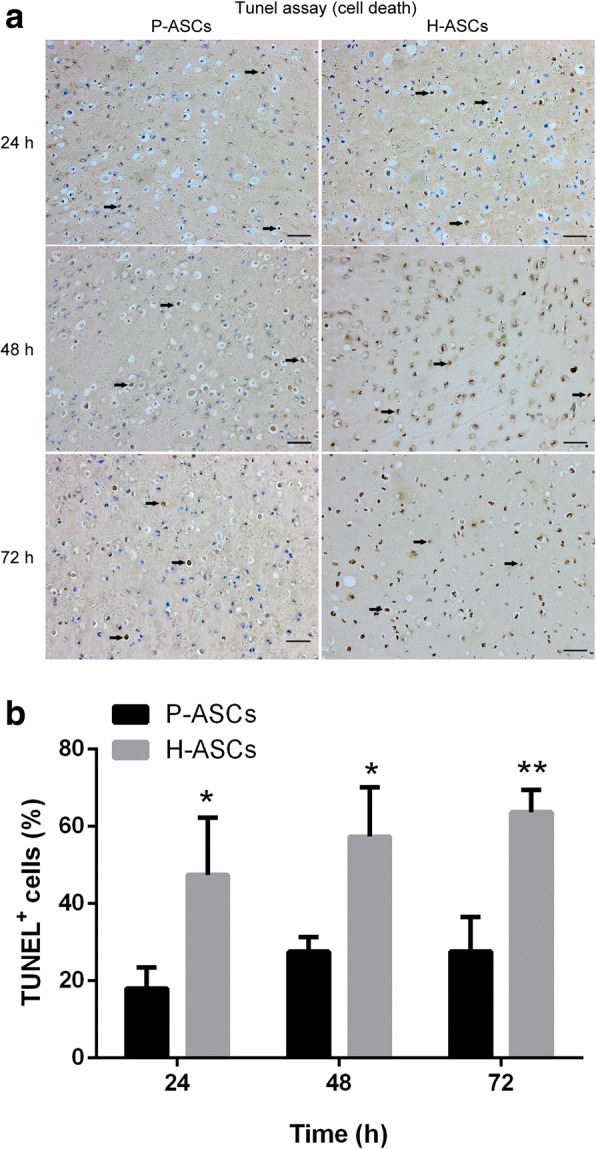
Physioxia increased ASC survivability in vivo. After mixing with 80 μL of fibrin gel, 1 × 10^6^ P-ASCs or H-ASCs were subcutaneously transplanted into the dorsum of nude mice. The implants were extracted after 24, 48, and 72 h. **a** TUNEL assay was used to stain the nucleus of dead cells. The *black arrows* indicate dead cells. **b** The TUNEL^+^ cell rate was determined by the ratio of TUNEL^+^ cells versus total cells. Three fields were quantified. Data are presented as the mean ± SD, *P < 0.05, **P < 0.01, Student’s *t* tests, scale bar = 100 μm. *ASCs* adipose-derived stem cells, *H-ASCs* hyperoxia ASCs, *P-ASCs* physioxia ASCs, *TUNEL* TdT-mediated dUTP-biotin nick end labeling

The number of dead cells 24, 48, and 72 h after implantation with fibrin gel was detected by TUNEL assay (Fig. 8a); compared to H-ASCs (47.46 ± 8.58%, 57.35 ± 7.41% and 63.70 ± 3.32%), P-ASCs (18.04 ± 3.13%, 27.56 ± 2.20% and 27.62 ± 5.13%) showed a significantly lower death rate (Fig. 8b).

## Discussion

Offering safe and effective cell therapy products for clinical applications is consistent with good manufacturing practice (GMP) guidelines, which should be followed during the entire process of isolating, expanding and transplanting ASCs [32]. The present study compared ASCs cultured under hyperoxia (20% O_2_) and physioxia (2% O_2_, oxygen concentration in situ) and provides compelling evidence that the latter could be a more effective approach owing to the advantages of retaining cell proliferation, migration, survival in ischemia and angiogenesis, and suppressing senescence and apoptosis.

There are no differences between P-ASCs and H-ASCs in terms of immunophenotype, morphology or adipogenesis, and a previous study [25] revealed that culturing ASCs under physioxia does not increase the risk of tumourigenesis associated with ASCs, indicating that P-ASCs are safe for clinical therapy. Physioxia promoted cell proliferation and migration, and many studies have attributed this effect to the stabilization of HIF-1 in the lack of O_2_ [33, 34]. However, the ROS level was also increased in P-ASCs, suggesting that transient physioxia can restore proliferation and migration through the augmentation of ROS [35]. Furthermore, we showed that without the injury caused by hyperoxia, physioxia is an appropriate condition for maintaining ASC proliferation and migration.

The relationship between physioxia and ROS is complicated [36]. In principle, HIF-1 decreases the ROS level [37, 38], which should be lower in P-ASCs, but the results show the opposite effect. The underlying mechanism remains unknown, especially in stem cells.

Many studies have shown the ability of HIF-1 to enhance angiogenesis under transient physioxia [39, 40], but consistent with most studies on physioxia and ASCs, the cells were isolated from a physioxic niche and then cultured under atmospheric hyperoxia, which could injure the bioactivity of the cells. Thus, the discrepancy of such bioactivity between P-ASCs and H-ASCs is not due to the acceleration of physioxia but reflects the damage caused by hyperoxia. Although transient physioxia preconditioning would be applied prior to transplantation for recovery, in the present method, culturing cells under physioxia through the entire in vitro period may be a better approach; however, further research is required.

To acquire an excellent stem cell product, cell viability should also be considered. Physioxia evidently suppressed senescence and apoptosis under nonstressful condition. The required survival of cells implanted in an ischemic environment composed of low oxygen, glucose, and pH levels is a main barrier for cell therapy [41]. Thus, we established an ischemic model and observed increased adaptability in P-ASCs; the same effect was observed in hypoxic, acidic, and nutrient-depleted environments, explaining the superiority of P-ASCs under these conditions and resulting in preferable adaptability in an ischemic environment.

The underlying mechanisms induced by HIF-1 and described in a previous study using an HIF-1 activator [31] were also observed in P-ASCs, but with an inverse trend in the ROS level. Briefly, more efficient aerobic oxidation (switch of cytochrome c oxidase subunit COX4I1 to COX4I2) and a switch to glycolysis (declined mitochondrial mass (Fig. 6a and b) caused by BNIP3 and increased glycolysis by PDK1 and LDHA) indicate adaptability to hypoxia (Fig. 5b). Additionally, enhanced glucose uptake (GLUT1 and GLUT3 (Fig. 7a and b)), glycogen synthesis (PGM and GYS1 (Fig. 7c)), and glycogen breakdown (PYGL) demonstrated cell adaptation to nutrient depletion (Fig. 5d), while an alkalescent intracellular pH (Fig. 6e) (CAR9, NHE2 and NHE3 [export H^+^] and MCT4 [export lactate] (Fig. 6d)) indicated adaptability to acidic conditions (Fig. 5c).

This study shows for the first time that culturing ASCs under physioxia for the entire in vitro term could induce metabolic alterations and improve ASC survival in ischemic environment. Our observations illustrate the molecular, cellular, and in vivo biological effects induced by physioxia in ASCs, presenting a significant mechanistic basis for culturing ASCs under physioxia for cell therapy. However, longer culture periods should be examined to guarantee the security of cell properties under this condition. Moreover, specific cell therapy models should be constructed to verify the ultimate efficacy of the cells, including when applied in adipose regeneration, heart failure treatment, and wound healing.

## Conclusions

In summary, the present results suggest that culturing ASCs under physioxia (2% O_2_) for the entire in vitro period, not under conventional hyperoxia (20% O_2_), could be a more effective approach for cell therapy applications owing to the improvements in proliferation, migration, survival and angiogenesis, and suppression of senescence and apoptosis.

## Abbreviations

2-NBDG: 2-[N-(7-nitrobenz-2-oxa-1,3-diazol-4-yl) amino]-2-deoxy-D-glucose
ASCs: Adipose-derived stem cells
BCECF-AM: 2′,7′-bis-(2-carboxyethyl)-5-(and-6)-carboxyfluorescein, acetoxymethyl ester
BHA: Butylated hydroxyanisole
BNIP3: BCL2/adenovirus E1B 19 kDa protein-interacting protein 3
CAR9: Carbonic anhydrase 9
COX4I2: Cytochrome c oxidase subunit 4 isoform 2
FBS: Fetal bovine serum
GLUT1: Glucose transporter 1
GLUT3: Glucose transporter 3
GMP: Good manufacturing practice
GYS1: Glycogen synthase 1
H_2_DCFDA: Dihydrodichlorofluorescein diacetate
HIF-1: Hypoxia-inducible factor 1
HRP: Horseradish peroxidase
IBMX: 3-isobutyl-1-methylxanthine
LDHA: Lactate dehydrogenase A
MCT4: Monocarboxylate transporter 4
NAO: Nonyl acridine orange
NHE2: Sodium-hydrogen exchanger 2
NHE3: Sodium-hydrogen exchanger 3
PAS: Periodic acid-Schiff (PAS)
PBS: Phosphate-buffered saline
PDK1: Pyruvate dehydrogenase kinase 1
PGM: Phosphoglucomutase
PVDF: Polyvinylidene fluoride
PYGL: Liver isoform of glycogen phosphorylase
RIPA: Radioimmunoprecipitation assay
ROS: Reactive oxygen species)
RT-PCR: Real-time polymerase chain reaction
SA-β-Gal: Senescence-associated β-galactosidase
TUNEL: TdT-mediated dUTP-biotin nick-end labeling
VEGF: Vascular endothelial growth factor
VEGF-R2: Vascular endothelial growth factor receptor 2
vWF: von Willebrand factor
WST-8: Cell Counting Kit 8

## References

[1] Rodbell M (1964). Metabolism of isolated fat cells. I. Effects of hormones on glucose metabolism and lipolysis. J Biol Chem.

[2] Nordberg RC, Loboa EG (2015). Our fat future: translating adipose stem cell therapy. Stem Cells Transl Med.

[3] Zuk PA, Zhu M, Mizuno H, Huang J, Futrell JW, Katz AJ (2001). Multilineage cells from human adipose tissue: implications for cell-based therapies. Tissue Eng.

[4] Kachgal S, Putnam AJ (2011). Mesenchymal stem cells from adipose and bone marrow promote angiogenesis via distinct cytokine and protease expression mechanisms. Angiogenesis.

[5] Zuk PA, Zhu M, Ashjian P, De Ugarte DA, Huang JI, Mizuno H (2002). Human adipose tissue is a source of multipotent stem cells. Mol Biol Cell.

[6] Melief SM, Zwaginga JJ, Fibbe WE, Roelofs H (2013). Adipose tissue-derived multipotent stromal cells have a higher immunomodulatory capacity than their bone marrow-derived counterparts. Stem Cells Transl Med.

[7] Qin JB, Li KA, Li XX, Xie QS, Lin JY, Ye KC (2012). Long-term MRI tracking of dual-labeled adipose-derived stem cells homing into mouse carotid artery injury. Int J Nanomedicine.

[8] Varghese J, Griffin M, Mosahebi A, Butler P (2017). Systematic review of patient factors affecting adipose stem cell viability and function: implications for regenerative therapy. Stem Cell Res Ther.

[9] Toyserkani NM, Jorgensen MG, Tabatabaeifar S, Jensen CH, Sheikh SP, Sorensen JA (2017). Concise review: a safety assessment of adipose-derived cell therapy in clinical trials: a systematic review of reported adverse events. Stem Cells Transl Med.

[10] Arrizabalaga JH, Nollert MU (2017). Properties of porcine adipose-derived stem cells and their applications in preclinical models. Adipocyte.

[11] Riis S, Zachar V, Boucher S, Vemuri MC, Pennisi CP, Fink T (2015). Critical steps in the isolation and expansion of adipose-derived stem cells for translational therapy. Expert Rev Mol Med.

[12] Naveiras O, Daley GQ (2006). Stem cells and their niche: a matter of fate. Cell Mol Life Sci.

[13] Spradling A, Drummond-Barbosa D, Kai T (2001). Stem cells find their niche. Nature.

[14] Kim WS, Han J, Hwang SJ, Sung JH (2014). An update on niche composition, signaling and functional regulation of the adipose-derived stem cells. Expert Opin Biol Ther.

[15] Ivanovic Z (2009). Hypoxia or in situ normoxia: The stem cell paradigm. J Cell Physiol.

[16] Mohyeldin A, Garzon-Muvdi T, Quinones-Hinojosa A (2010). Oxygen in stem cell biology: a critical component of the stem cell niche. Cell Stem Cell.

[17] Anderson DE, Markway BD, Weekes KJ, HE MC, Johnstone B (2018). Physioxia promotes the articular chondrocyte-like phenotype in human chondroprogenitor-derived self-organized tissue. Tissue Eng A.

[18] Prabhakar NR, Semenza GL (2015). Oxygen sensing and homeostasis. Physiology (Bethesda).

[19] Hubbi ME, Semenza GL (2015). Regulation of cell proliferation by hypoxia-inducible factors. Am J Physiol Cell Physiol.

[20] Hawkins KE, Sharp TV, McKay TR (2013). The role of hypoxia in stem cell potency and differentiation. Regen Med.

[21] Buravkova LB, Andreeva ER, Gogvadze V, Zhivotovsky B (2014). Mesenchymal stem cells and hypoxia: where are we?. Mitochondrion.

[22] Fotia C, Massa A, Boriani F, Baldini N, Granchi D (2015). Prolonged exposure to hypoxic milieu improves the osteogenic potential of adipose derived stem cells. J Cell Biochem.

[23] Qin HH, Filippi C, Sun S, Lehec S, Dhawan A, Hughes RD (2015). Hypoxic preconditioning potentiates the trophic effects of mesenchymal stem cells on co-cultured human primary hepatocytes. Stem Cell Res Ther.

[24] Yu Y, Zhou Y, Cheng T, Lu X, Yu K, Hong J (2016). Hypoxia enhances tenocyte differentiation of adipose-derived mesenchymal stem cells by inducing hypoxia-inducible factor-1alpha in a co-culture system. Cell Prolif.

[25] Choi JR, Pingguan-Murphy B, Wan Abas WA, Yong KW, Poon CT, Noor Azmi MA (2015). In situ normoxia enhances survival and proliferation rate of human adipose tissue-derived stromal cells without increasing the risk of tumourigenesis. PLoS One.

[26] Choi JR, Pingguan-Murphy B, Wan Abas WA, Noor Azmi MA, Omar SZ, Chua KH (2014). Impact of low oxygen tension on stemness, proliferation and differentiation potential of human adipose-derived stem cells. Biochem Biophys Res Commun.

[27] Wan Safwani WKZ, Choi JR, Yong KW, Ting I, Mat Adenan NA, Pingguan-Murphy B (2017). Hypoxia enhances the viability, growth and chondrogenic potential of cryopreserved human adipose-derived stem cells. Cryobiology.

[28] Yamamoto Y, Fujita M, Tanaka Y, Kojima I, Kanatani Y, Ishihara M (2013). Low oxygen tension enhances proliferation and maintains stemness of adipose tissue-derived stromal cells. BioResearch open access.

[29] Fotia C, Massa A, Boriani F, Baldini N, Granchi D (2015). Hypoxia enhances proliferation and stemness of human adipose-derived mesenchymal stem cells. Cytotechnology.

[30] Liu W, Zhou L, Zhou C, Zhang S, Jing J, Xie L (2016). GDF11 decreases bone mass by stimulating osteoclastogenesis and inhibiting osteoblast differentiation. Nat Commun.

[31] Chen C, Tang Q, Zhang Y, Dai M, Jiang Y, Wang H, et al. Metabolic reprogramming by HIF-1 activation enhances survivability of human adipose-derived stem cells in ischaemic microenvironments. Cell Prolif. 2017;50 10.1111/cpr.12363 PMC6529110 28752896

[32] Giancola R, Bonfini T, Iacone A (2012). Cell therapy: cGMP facilities and manufacturing. Muscles Ligaments Tendons J.

[33] Lee HJ, Ryu JM, Jung YH, Oh SY, Lee SJ, Han HJ (2015). Novel pathway for hypoxia-induced proliferation and migration in human mesenchymal stem cells: involvement of HIF-1alpha, FASN, and mTORC1. Stem Cells.

[34] Kakudo N, Morimoto N, Ogawa T, Taketani S, Kusumoto K (2015). Hypoxia enhances proliferation of human adipose-derived stem cells via HIF-1a activation. PLoS One.

[35] Kim JH, Park SH, Park SG, Choi JS, Xia Y, Sung JH (2011). The pivotal role of reactive oxygen species generation in the hypoxia-induced stimulation of adipose-derived stem cells. Stem Cells Dev.

[36] Gorlach A, Dimova EY, Petry A, Martinez-Ruiz A, Hernansanz-Agustin P, Rolo AP (2015). Reactive oxygen species, nutrition, hypoxia and diseases: Problems solved?. Redox Biol.

[37] Horak P, Crawford AR, Vadysirisack DD, Nash ZM, DeYoung MP, Sgroi D (2010). Negative feedback control of HIF-1 through REDD1-regulated ROS suppresses tumorigenesis. Proc Natl Acad Sci U S A.

[38] Semenza GL (2009). Regulation of oxygen homeostasis by hypoxia-inducible factor 1. Physiology (Bethesda).

[39] Fan L, Zhang C, Yu Z, Shi Z, Dang X, Wang K (2015). Transplantation of hypoxia preconditioned bone marrow mesenchymal stem cells enhances angiogenesis and osteogenesis in rabbit femoral head osteonecrosis. Bone.

[40] Srinivasan S, Chitalia V, Meyer RD, Hartsough E, Mehta M, Harrold I (2015). Hypoxia-induced expression of phosducin-like 3 regulates expression of VEGFR-2 and promotes angiogenesis. Angiogenesis.

[41] Rey S, Luo W, Shimoda LA, Semenza GL (2011). Metabolic reprogramming by HIF-1 promotes the survival of bone marrow-derived angiogenic cells in ischemic tissue. Blood.

